# Cloud-driven modulations of Greenland ice sheet surface melt

**DOI:** 10.1038/s41598-019-46152-5

**Published:** 2019-07-17

**Authors:** Masashi Niwano, Akihiro Hashimoto, Teruo Aoki

**Affiliations:** 1 Meteorological Research Institute, Japan Meteorological Agency, Tsukuba, 305-0052 Japan; 2 National Institute of Polar Research, Tachikawa, 190-8518 Japan

**Keywords:** Atmospheric science, Cryospheric science

## Abstract

Clouds have been recognized to enhance surface melt on the Greenland Ice Sheet (GrIS). However, quantitative estimates of the effects of clouds on the GrIS melt area and ice-sheet-wide surface mass balance are still lacking. Here we assess the effects of clouds with a state-of-the-art regional climate model, conducting a numerical sensitivity test in which adiabatic atmospheric conditions as well as zero cloud water/ice amounts are assumed (i.e., clear-sky conditions), although the precipitation rate is the same as in the control all-sky simulation. By including or excluding clouds, we quantify time-integrated feedbacks for the first time. We find that clouds were responsible for a 3.1%, 0.3%, and 0.7% increase in surface melt extent (of the total GrIS area) in 2012, 2013, and 2014, respectively. During the same periods, clouds reduced solar heating and thus daily runoff by 1.6, 0.8, and 1.0 Gt day^−1^, respectively: clouds did not enhance surface mass loss. In the ablation areas, the presence of clouds results in a reduction of downward latent heat flux at the snow/ice surface so that much less energy is available for surface melt, which highlights the importance of indirect time-integrated feedbacks of cloud radiative effects.

## Introduction

The Greenland ice sheet has lost a significant mass of ice since the early 1990’s^1–3^. Changes in the ice sheet mass (mass balance, MB) are a function of the surface mass balance (SMB) and ice discharge across the grounding line (D), where the ice starts to float: MB = SMB – D. Before the 2000s, the absolute values of the rates at which SMB was decreasing and discharge was increasing were almost the same^4^. Recently, however, SMB has played a dominant role in the GrIS’s negative mass balance with more than 80% of mass loss attributed to increased surface runoff^3,5–7^, which is defined as water that flows away from the ice sheet into the surrounding ocean. SMB is a consequence of energy and mass interactions between the atmosphere and the snow or ice surface. The lowest annual GrIS SMB since the 1990s, attributable largely to a record surface melt event^8^, was recorded in 2012^3,7^. Moreover, clouds played a key role in this event^9,10^ through the cloud radiative effect (CRE)^11–17^:

1 $${\rm{C}}{\rm{R}}{\rm{E}}={({S}_{{\rm{n}}{\rm{e}}{\rm{t}}}+{L}_{{\rm{n}}{\rm{e}}{\rm{t}}})}_{{\rm{a}}{\rm{l}}{\rm{l}}-{\rm{s}}{\rm{k}}{\rm{y}}}-{({S}_{{\rm{n}}{\rm{e}}{\rm{t}}}+{L}_{{\rm{n}}{\rm{e}}{\rm{t}}})}_{{\rm{c}}{\rm{l}}{\rm{e}}{\rm{a}}{\rm{r}}-{\rm{s}}{\rm{k}}{\rm{y}}}\,,$$

where *S*_net_ and *L*_net_ are net shortwave and longwave radiant fluxes at the snow/ice surface; *S*_net_ and *L*_net_ are taken as positive when they are directed into the surface. Typically, at the surface, the shortwave CRE (*S*_net, all-sky_ − *S*_net, clear-sky_) is negative whereas the longwave CRE (*L*_net, all-sky_ − *L*_net, clear-sky_) is positive. In polar regions covered with snow, which has a very high albedo, the longwave CRE is sometimes greater than the shortwave CRE (CRE is positive). This situation is known as the “radiation paradox^11^” where by surface heating can occur even under cloudy-sky conditions, so clouds act as a forcing factor on the climate system.

Although the radiation paradox^11^ is qualitatively valid, quantitative impacts of both the CRE and its resultant feedback processes on the changes in (not short time event scale but) climatic ice sheet physical conditions, namely ice sheet-wide surface melt and the resultant SMB have not yet been fully evaluated. Recently, one study^18^, which examined climatic trends for cloud fraction and the ice sheet SMB simulated by a regional climate model, found a relation: a decreasing cloud fraction has driven an ice sheet SMB reduction since around 1995. However, the quantitative impacts of clouds, as well as the underlying physical mechanisms by clouds on surface melting have not been sufficiently understood to rank their effects relative to other melt processes, although the importance of changes in the atmospheric circulation pattern over the ice sheet especially during the summer has been highlighted^18^. Here, we used the regional climate model NHM–SMAP^19^ to perform a model sensitivity test investigating the role and effects of clouds in which atmospheric conditions were assumed to be adiabatic as well as zero cloud water/ice amounts, thus eliminating the CRE (see Methods for more detail). Then by comparing the results between control (all-sky) and sensitivity (clear-sky) simulations, we evaluated the quantitative effects of clouds on the ice sheet surface melt area extent and SMB from 2011–2014. Our method has an advantage in that it allows us to consider time-integrated atmospheric feedback processes imposed by instantaneous changes in downward radiative properties caused by the presence or absence of clouds; these time-integrated feedback processes were not taken into account by any previous studies.

## Cloud fraction and CRE

**Figure 1:**
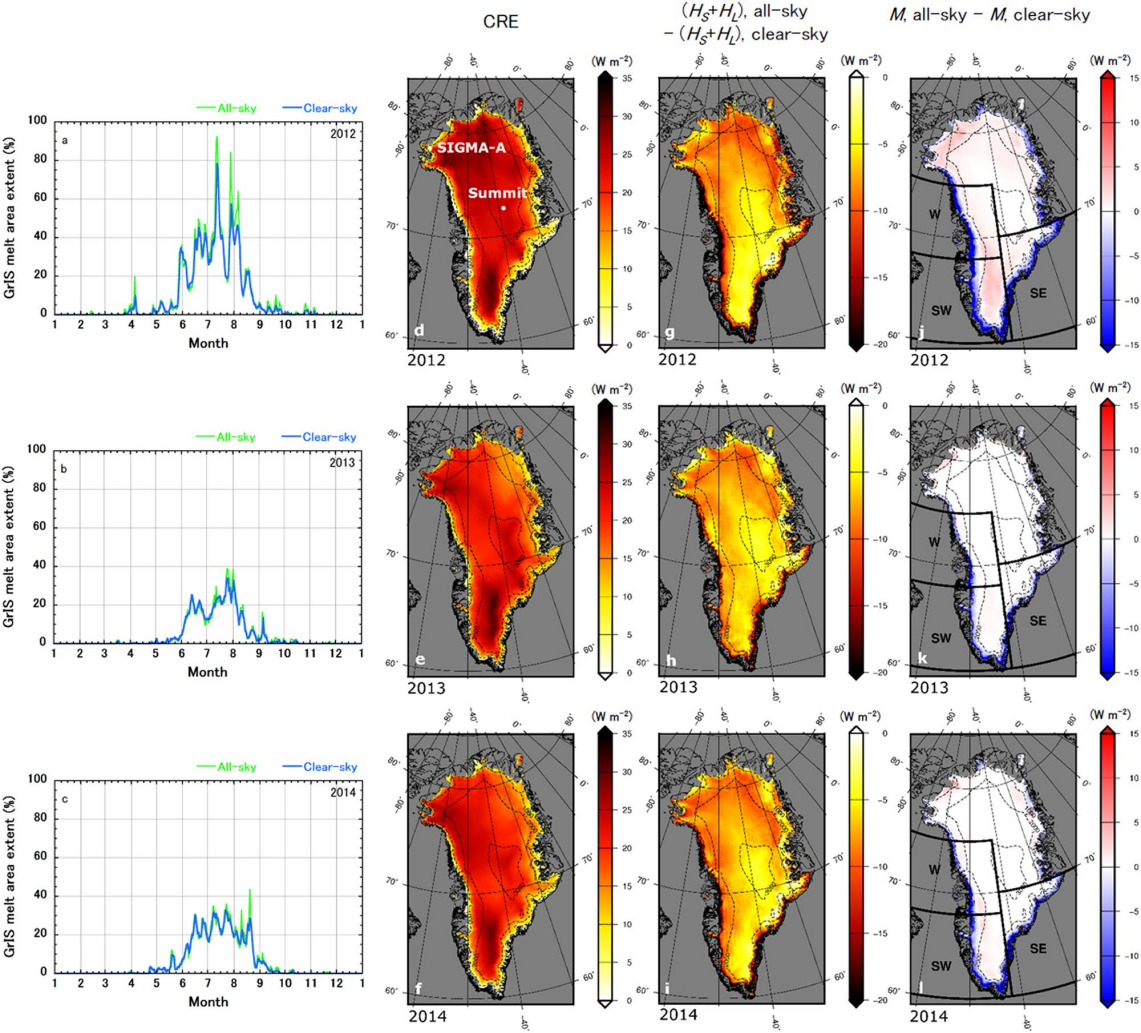
Impacts of clouds on the ice sheet surface melt area extent and on SEB simulated during JJA by NHM–SMAP. (**a**–**c**), Temporal changes in the ice sheet-wide surface melt area extent in 2012, 2013, and 2014, respectively, for the all-sky and clear-sky cases. (**d**–**f**), Cloud radiative effects; (**g**–**i**), changes in turbulent heat fluxes; and (**j**–**l**), changes in the surface melt energy during JJA due to the presence of clouds in 2012, 2013, and 2014, respectively. All SEB values were obtained by subtracting the clear-sky simulation results from the all-sky simulation results. Contours on the ice sheet and peripheral ice caps indicate surface elevation (contour interval 1000 m). *In situ* measurement stations mentioned in this paper are indicated in panel d. The southeast (SE), west (W), and southwest (SW) drainage regions of the ice sheet are shown in panels j, k, and l.

Previously reported model validation results^19^ showed that NHM–SMAP successfully reproduced measured GrIS climate conditions and diurnal variations during our study period (2011–2014), which includes the 2012 record surface melt event^8^. We introduce here the concept of “mass balance year^20^”, defined as from September of the first year to August of the following year, because it is needed to calculate annual accumulated and averaged values. Simulated cloud fractions for the 2011–2012, 2012–2013, and 2013–2014 mass balance years are listed in Table 1, Supplementary Tables 1, 2, respectively. Overall, the annual average cloud fraction above the entire ice sheet was around 0.5, but the cloud fraction averaged over the summer (June, July, and August; JJA) was relatively low (around 0.45). The area-averaged value of the simulated annual average ice sheet CRE during each of the three mass balance years (Supplementary Fig. 1) was around 20 W m^−2^. In the northern part of the ice sheet, as well as in low-elevation areas, the CRE was relatively small. Low albedo mainly due to the exposure of bare ice in the low-elevation areas, where most melt and runoff occur, obviously affected the low CRE, because low albedo enhances the shortwave CRE. Cloud height above the surface might also affect the CRE, which should be investigated further in the future. At Summit station (72.68°N, 38.58°W; see Fig. 1), the CRE was estimated to be 33 W m^−2^ from January 2011 to October 2013 by a radiative transfer model that used *in situ* measurements^21^. The NHM–SMAP-simulated CRE at Summit station from September 2011 to October 2013 was lower (25 W m^−2^). However, comparison of CREs calculated by different techniques requires careful consideration^21^. The observation-based study mentioned above^21^ considered only instantaneous changes in downward radiant fluxes, whereas in the present study, we considered additional atmospheric feedbacks, which might account for the different result. We discuss the validity of the atmospheric responses of the clear-sky model sensitivity test conducted with NHM–SMAP in the last section of this paper.

**Table 1:** 2011–2012 area-averaged cloud fraction and cloud effects on the ice sheet SEB, SMB, and surface meteorological conditions calculated by NHM–SMAP. *P*, *T*, and *q* represent surface pressure, 2 m air temperature, and 2 m water vapour mixing ratio, respectively (w.e. means “water equivalent”).

	Entire ice sheet			Southern and western ablation areas		
	1112_MAM	1112_JJA	1112	1112_MAM	1112_JJA	1112
Cloud fraction	0.50	0.46	0.54	0.48	0.39	0.46
CRE (W m^2^)	19.0	18.8	20.8	19.3	−2.3	16.2
Δ*S*_net_ (W m^−2^)	−5.3	−12.0	−4.7	−9.0	−23.1	−8.8
Δ*L*_net_ (W m^−2^)	24.3	30.7	25.5	28.3	20.8	25.0
Δ*H*_S_ (W m^−2^)	−1.8	0.3	−2.0	0.4	0.3	1.1
Δ*H*_L_ (W m^−2^)	−3.4	−8.8	−5.0	−6.2	−24.1	−13.1
Δ*M* (W m^−2^)	0.0	−2.1	−0.5	−0.8	−26.3	−6.8
ΔRU (mm w.e. day^−1^)	−0.02	−0.91	−0.22	−0.42	−7.46	−1.82
ΔSU_*s*_ (mm w.e. day^−1^)	0.02	0.11	0.04	0.11	0.32	0.21
ΔSU_*ds*_ (mm w.e. day^−1^)	0.01	0.01	0.02	0.00	0.00	0.04
Δ*P* (hPa)	−0.3	−0.3	−0.2	−0.6	−0.2	−0.6
Δ*T* (K)	2.4	1.3	2.4	0.5	−0.2	0.6
Δ*q* (g kg^−1^)	0.1	−0.1	0.0	−0.1	−0.9	−0.3

All values except those for cloud fraction were obtained by subtracting the clear-sky simulation results from the all-sky simulation results. Each result is area-averaged over the entire ice sheet as well as over the low-elevation (<1000 m a.s.l.) ablation areas of the southern and western regions of the ice sheet (see Fig. 1) during March–May 2012 (1112_MAM), June–August 2012 (1112_JJA), and September 2011 to August 2012 (1112).

## Surface melt area extent

**Figure 2:**
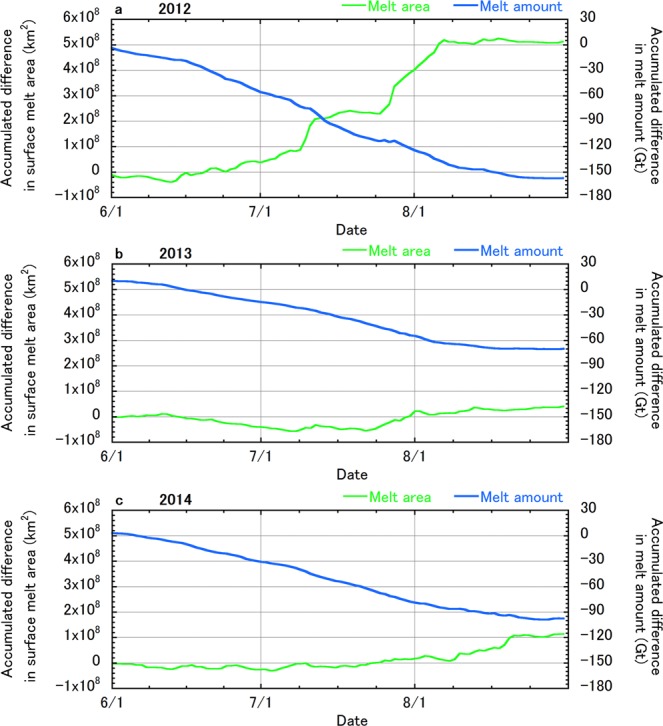
Enhanced ice sheet surface melt area and melt amount due to clouds during JJA simulated by NHM–SMAP. (**a**–**c**), Differences in ice sheet-wide accumulated surface melt area and accumulated melt amount with respect to 1 June between all-sky and clear-sky simulations during 2012, 2013, and 2014, respectively. These properties were obtained by subtracting the clear-sky accumulated surface melt area and melt amount from those of the all-sky simulation.

Comparison of the ice sheet surface melt area extents in 2012, 2013, and 2014 between the all-sky and clear-sky NHM–SMAP simulations (Fig. 1a–c) illustrates that the presence of clouds played a role in enhancing the surface melt area extent, especially during summer, in every year. The difference in the ice sheet melt area extent due to clouds during JJA in 2012, 2013, and 2014 was 3.1%, 0.3%, and 0.7% of the total ice sheet area, respectively; thus, clouds had an exceptionally large effect on the GrIS surface melt in 2012. This result is attributable to warm air associated with the record North American heat wave, the transport of water vapour via an atmospheric river over the Atlantic Ocean to Greenland, and the presence of anomalously warm ocean waters south of Greenland observed in summer 2012^22^. Accumulated melt areas during JJA were enhanced by the presence of clouds by 5.1 × 10^8^, 4.2 × 10^7^, and 1.2 × 10^8^ km^2^, respectively (Fig. 2). However, total melt during the periods decreased by 157, 70, and 97 Gt, respectively due to the presence of clouds (Fig. 2).

The simulated CRE during JJA (Fig. 1d–f) of these periods was high over the inland area of the ice sheet but very low in the ablation areas, where most meltwater production and runoff occurs. Another surface energy balance (SEB) component (see Methods for the definition of SEB), however, was indirectly but significantly affected by the presence or absence of clouds: Turbulent heat fluxes (sensible and latent heat fluxes; see Methods) differed depending on whether clouds were present during JJA in 2012, 2013, and 2014 (Fig. 1g–i), and the changes were particularly large in the low-elevation (<1000 m a.s.l.) ablation areas of the southeast, west, and southwest regions of the ice sheet. As a result, JJA surface melt energy (see Methods) was increased on the inland ice sheet by the CRE (i.e., surface melt area extent was enhanced under all-sky conditions), whereas it was decreased in the ice sheet ablation areas by the indirect CRE (more surface melt could occur under clear-sky conditions) (Fig. 1j–l). For reference, the NHM–SMAP-simulated all-sky SEB results for JJA are displayed in Supplementary Figure 2.

Here, we define the low-elevation areas (less than 1000 m a.s.l.) of the southeast, west, and southwest ice sheet shown in Fig. 1 as the ablation areas of the southern and western regions of the GrIS. The area-averaged cloud fractions during the study period were lower over the ablation areas of the southern and western regions of the GrIS compared to those over the entire ice sheet (Table 1, Supplementary Tables 1, 2). Although the relatively small CRE in low-elevation areas can be attributed to low albedo in those areas, the lower cloud fraction might also have led to a smaller CRE. Quantitative impacts of cloud fraction on the ice sheet ablation area CRE is beyond the scope of this study; however, it should be examined further in the future. Moreover, the main contributor to the changes in net turbulent heat flux in the ablation areas of the southern and western regions of the GrIS was the latent heat flux (17.3 to 24.1 W m^−2^; Table 1 and Supplementary Tables 1, 2). This result highlights the fact that for a comprehensive understanding of the role of clouds in the climate system around the ice sheet, it is not sufficient to examine only instantaneous changes in downward radiant fluxes induced by the presence or absence of clouds; it is also necessary to consider indirect time-integrated atmospheric feedbacks caused by the CRE. We discuss the cause of the large changes in latent heat flux during JJA in the ablation areas of the southern and western regions of the GrIS in relation to the presence or absence of clouds in the last section of this paper.

## Surface mass balance

**Figure 3:**
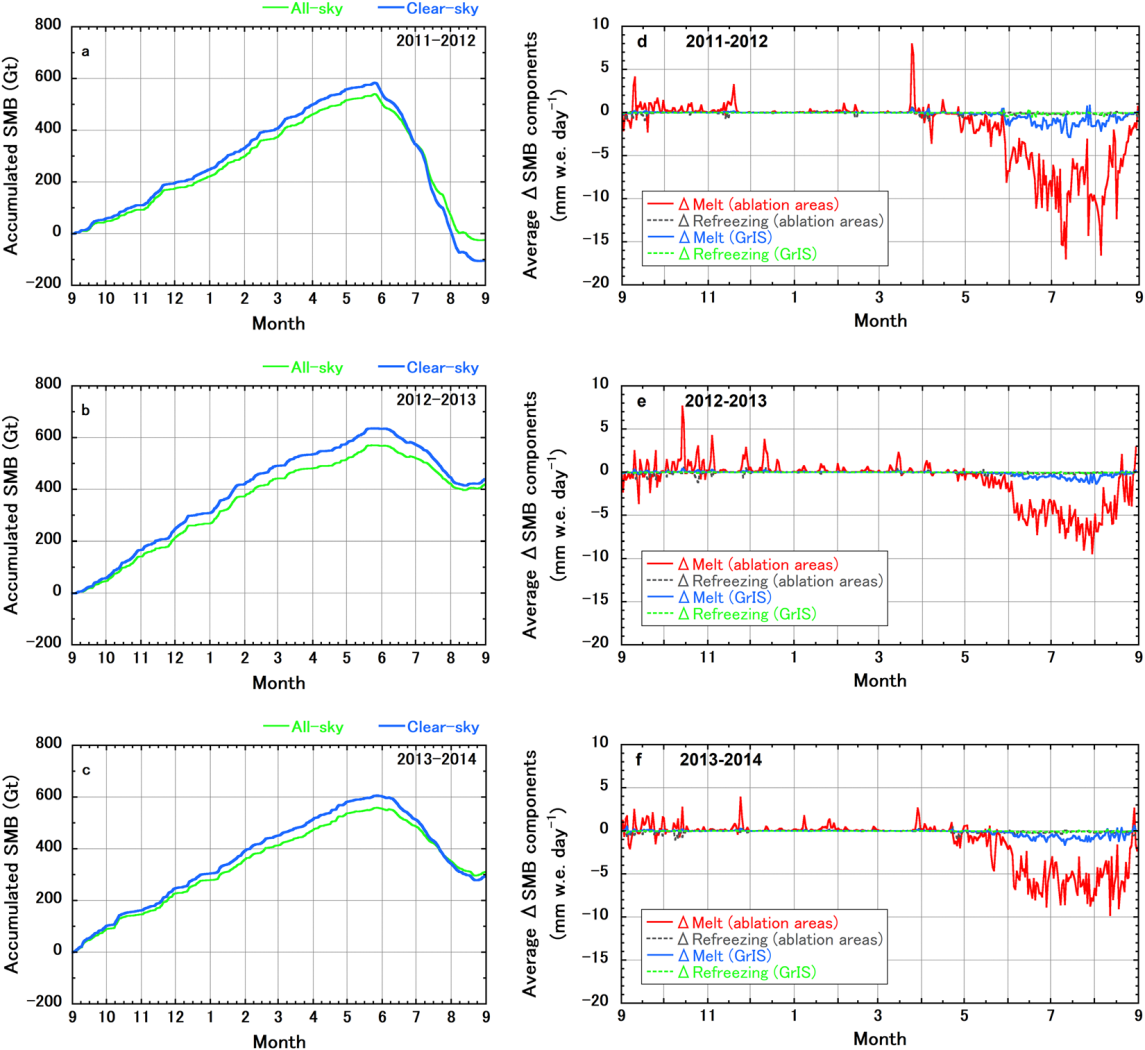
Impacts of clouds on the ice sheet-wide SMB simulated by NHM–SMAP. (**a**–**c**) Temporal evolution of the accumulated ice sheet SMB during the three mass balance years (2011–2012, 2012–2013, and 2013–2014) under all-sky and clear-sky conditions. (**d**–**f**) Temporal changes in the daily differences in melt and refreezing rates caused by the presence of clouds averaged over the ablation areas of the southern and western regions of the ice sheet as well as the entire ice sheet during the 2011–2012, 2012–2013, and 2013–2014 mass balance years; all values were obtained by subtracting clear-sky simulation results from all-sky simulation results.

Contrary to the surface melt area extent result, comparisons of the annual accumulated ice sheet SMB within a mass balance year between the all-sky and clear-sky NHM–SMAP simulations show exactly the opposite impact of the CRE (Fig. 3a–c): The annual accumulated ice sheet SMB at the end of August simulated under clear-sky conditions was less positive than (2011–2012), or almost equal (2012–2013 and 2013–2014), to that simulated under all-sky conditions, although the winter to spring accumulation rates under all-sky conditions were lower than those under clear-sky conditions during the entire study period. Here, note that the same spatial and temporal precipitation rate patterns were used in the sensitivity test as in the control all-sky simulation, because our focus here was on only the CRE and its resultant atmospheric feedbacks. The reason why the above-mentioned differences in the winter to spring accumulation rates were made is discussed later in this section.

Analyses of each SMB component (see Methods) showed that surface sublimation during the winter to spring accumulation period was enhanced under all-sky conditions (see Table 1 and Supplementary Tables 1, 2; the difference during March, April, and May [MAM] of each year was 0.02 mm water equivalent [w.e.] day^−1^). This result can be attributed to the difference in the simulated MAM surface air temperature due to the presence or absence of clouds; simulated MAM surface air temperatures over the entire ice sheet under all-sky conditions were higher by more than 2 K than those under clear-sky conditions in each year (Table 1 and Supplementary Tables 1, 2). Note, however, that because air temperatures were still very low during MAM, the energy available for surface melt, was not affected by the difference between all-sky and clear-sky conditions. During JJA of 2012, 2013, and 2014, clouds played a role in reducing daily area-averaged runoff from the ice sheet by 0.91, 0.45, and 0.57 mm w.e. day^−1^ (ice sheet-integrated runoff of 1.6, 0.8, and 1.0 Gt day^−1^), respectively (Table 1 and Supplementary Tables 1, 2).

In general, more refreezing can occur under clear-sky conditions, mainly because of night-time longwave cooling, than under all-sky conditions. Therefore, we investigated melt and refreezing rates during the study period (Fig. 3d–f; see also Table 1 and Supplementary Tables 1, 2) and found changes in these SMB-related area-averaged components due to the presence or absence of clouds for both the ablation areas of the southern and western regions of the GrIS and the entire ice sheet. These results indicate that refreezing in the ablation areas of the southern and western regions of the GrIS was not enhanced even under clear-sky conditions, because the higher latent heat flux under clear-sky conditions prevented surface cooling even at night in the ablation areas of the southern and western regions of the GrIS.

## Discussion

We investigated the validity of the atmospheric responses in a clear-sky model sensitivity test, as well as the cause of the large changes in latent heat flux during JJA in the ablation areas of the southern and western regions of the GrIS depending on the presence or absence of clouds. For this purpose, we examined changes in surface pressure, air temperature, the water vapour mixing ratio, and wind speed attributable to clouds.

During JJA, surface pressure around the ice sheet was higher under clear-sky conditions (Supplementary Fig. 3a–c; also see Table 1, and Supplementary Tables 1, 2). Latent heating in the atmosphere has been recognized to play an important role in reducing surface pressure; for example, rapid intensification of the *Queen Elizabeth II* storm, an extratropical cyclone that developed in September 1978, was attributed mainly to diabatically-induced processes including latent heating, which induced a positive feedback between adiabatic and diabatic movements^23^. In addition, such a numerical experiment with the atmospheric part of NHM–SMAP (JMA–NHM^24^) has ever been conducted^25^. Therefore, we judge the qualitative atmospheric responses induced by the absence of clouds simulated in this study to be robust. In general, the surface pressure system over the ice sheet is considered to be strongly related to the general circulation pattern (the North Atlantic Oscillation index is a useful indicator of the relationship)^22,26–29^. Our results suggest that atmospheric latent heating due to cloud formation over the ice sheet has a great effect on the general circulation pattern in the area.

JJA inland surface air temperature was simulated to be higher under the all-sky conditions (Supplementary Figures 3d–f, also see Table 1 and Supplementary Tables 1, 2); this result can be attributed mainly to the enhancement of inland surface melt under the all-sky conditions reported above; however, because the changes in surface air temperature were very low in the ablation areas, it cannot account for the changes in latent heat flux there.

On the other hand, increased surface moisture was simulated in the ablation areas under clear-sky conditions (Supplementary Fig. 3g–i, also see Table 1 and Supplementary Tables 1, 2). Once a high-pressure system has developed over the ice sheet because of the absence of clouds, moisture transport from the lower atmosphere to the upper atmosphere is restrained; as a result, moisture is trapped in the low-elevation ablation areas around the ice sheet and downward latent heat flux there is increased. These results account for the higher latent heat flux during JJA in the ablation areas of the southern and western regions of the GrIS under clear-sky conditions.

Although it is well known that surface wind speed also affects surface turbulent heat fluxes, we found that JJA average changes in surface wind speed associated with the presence or absence of clouds over the ablation areas of the southern and western regions of the GrIS were very low during the study period (0.03, –0.19, and –0.09 m s^−1^ for 2011–2012, 2012–2013, and 2013–2014, respectively). However, changes in surface wind speed due to the presence or absence of clouds are relatively high especially in the northern GrIS (Supplementary Fig. 3j–l), suggesting that the response of katabatic winds to cloud condition is relatively strong in the northern GrIS.

In this paper, we did not discuss the effects of light-absorbing impurities such as black carbon and dust^30^, as well as biological materials like cryoconite^31^ on the CRE and its resultant feedback processes. Although detailed investigation of the effects is beyond the scope of this paper, we suppose the CRE and its resultant feedback can be restrained due to the presence of light-absorbing impurities, because these reduce snow and ice albedo, and thus the absolute value of shortwave CRE becomes larger.

## Methods

We applied a state-of-the-art physically based spatially and temporally high-resolution regional climate model called NHM–SMAP (Non-Hydrostatic atmospheric Model with the Snow Metamorphism and Albedo Process model) to the GrIS^19^. The model configuration for the control simulation (all-sky simulation) in the present study is exactly the same as the original configuation^18^. In the atmospheric part of NHM–SMAP (JMA-NHM^24^; Japan Meteorological Agency Non-Hydrostatic atmospheric Model), we employed the “weather forecast mode”, in which the atmospheric profile is initialized every day by referring to parent reanalysis data (NHM–SMAP boundary conditions; in this study, the Japanese 55-year reanalysis JRA-55^32^ dataset) to prevent large deviations between the parent (JRA-55) and NHM–SMAP atmospheric fields. In the weather forecast mode calculations, a 30-h-long simulation was carried out every day, starting from 18:00 UTC of the previous day; model outputs from the initial 6 h spin-up period were discarded, and model outputs from the last 24 h were used. In JMA-NHM, the improved Mellor-Yamada Level 3 turbulence closure boundary layer scheme^33^ is used to couple planetary boundary layer and free atmosphere.

The initial snow/firn/ice physical conditions for the entire ice sheet on 1 September 2011 were prepared by performing a 30year spin-up of the NHM-SMAP model^19^. After that, no initialization of the snow/ice part of the model (physical snowpack model of SMAP^34,35^) was made (“climate simulation mode”) during the calculation period, because no information useable for such an initialization was available.

The SMAP model incorporates the physically based snow albedo model^36^ in which snow albedo and the solar heating profile in the snowpack are calculated by explicitly considering the effects of snow grain size, light-absorbing impurity (LAI) concentrations in snow/ice, the cloud fraction, and solar conditions. The present study did not take account of LAI (pure snow was assumed), following the original configuration of NHM–SMAP applied to the GrIS^19^.

Various aspects of NHM–SMAP were validated for the GrIS from 2011–2014, the same as the study period in the present study, by utilizing *in situ* measurements^19^. The validation results demonstrated that the model successfully reproduced measured features of the GrIS climate and diurnal variations during that period^19^.

The model diagnosed daily melt area extent from hourly snow, firn, and ice surface temperature data and water content profiles. First, the daily maximum surface temperature was extracted at each grid point. If the value reached 273.15 K and the top model layer contained water at the time when the maximum surface temperature was recorded, the grid point was considered to have undergone surface melt^19^.

The ice sheet SMB is calculated by NHM–SMAP as follows^19^:

2 $${\rm{SMB}}=P-{{\rm{SU}}}_{s}-{{\rm{SU}}}_{ds}-\mathrm{RU},$$

where *P* is precipitation, SU_*s*_ is sublimation or evaporation from the surface, SU_*ds*_ is sublimation from drifting snow particles, and RU is runoff. RU is governed mainly by the balance between melt and refreezing rates in the surface snow or ice layer.

In numerical investigations of the effects of clouds on the changes in atmospheric field, it is not sufficient to modify only atmospheric radiation, because the presence or absence of clouds affects not only atmospheric radiation but also latent heating in the atmosphere (temperature profile in the atmosphere)^37^. According to a previous study^38^, the interaction between clouds and circulation primarily results from three processes: phase changes, radiative transfer, and turbulent transport of air parcels, where condensation and evaporation processes associated with the formation, the maturation or the dissipation of clouds, and the interaction of clouds with solar and infrared radiation, lead to atmospheric heating and cooling perturbations, which stimulate waves and turbulence and which affect the horizontal and vertical distributions of temperature on a wide range of scales. Therefore, in the clear-sky numerical sensitivity test with NHM–SMAP performed in this study, we omitted latent heating in the atmosphere (i.e., condensation heating is set to zero; an adiabatic simulation was conducted) to account for the impacts of phase change, and also set cloud water/ice amounts to zero to assess the impacts of radiative transfer in the atmosphere. By omitting latent heating, the temperature lapse rate in the atmosphere does not follow the moist adiabatic lapse rate but always follows the dry adiabatic lapse rate. The dry adiabatic lapse rate (9.8 °C km^−1^) is higher than the moist adiabatic lapse rate because the latter is a result of latent heat release by the cloud formation. By setting cloud water/ice amounts to zero, clear-sky downward shortwave and longwave radiant fluxes can be calculated. At the same time, the model calculates changes in turbulent transport of air parcels in response to changes in the temperature lapse rate and downward radiant fluxes. In general, under the clear-sky condition, upward and downward vertical motions are expected to be suppressed, which would result in higher surface pressure^23^. Consideration of all the cloud-atmosphere interaction processes^38^ in the numerical sensitivity experiment is a significant advantage of the present study. However, the same spatial and temporal precipitation rate patterns were used in the sensitivity test as in the control all-sky simulation, because our focus here was on only the CRE and its resultant atmospheric feedbacks.

As mentioned above, temporal evolution of atmospheric fields was calculated in weather forecast mode in the control simulation. This model setting was also used in the sensitivity test; therefore, deviations in the atmospheric fields between the all-sky and clear-sky simulations are not expected to be large. Clouds are known as a “fast process” in the terrestrial climate system; therefore, examining the results of the clear-sky numerical sensitivity test on the weather forecast mode time scale can provide insights into the possible atmospheric responses to changes in cloud fraction not only over a period of several days but also on a much longer time scale^39,40^.

In the sensitivity test, profiles of snow and ice physical conditions were reset at the beginning of the 2011–2012, 2012–2013, and 2013–2014 mass balance years by referring to the control simulation data. As a result, feedbacks with a timescale of more than a year are beyond the scope of this study. An example comparison between the all-sky and clear-sky downward shortwave and longwave radiant fluxes calculated by NHM–SMAP for the SIGMA-A site, northwest GrIS (see Fig. 1d), during 10 to 15 July 2012 is displayed together with *in situ* measurements^10^ in Supplementary Fig. 4.

To understand simulated changes in the ice sheet melt area extent and SMB in detail, we introduce the concept of the snow and ice surface energy balance (SEB), defined as follows:

3 $${S}_{{\rm{n}}{\rm{e}}{\rm{t}}}+{L}_{{\rm{n}}{\rm{e}}{\rm{t}}}+{H}_{{\rm{S}}}+{H}_{{\rm{L}}}+{H}_{{\rm{R}}}+{H}_{{\rm{G}}}=M$$

where *S*_net_ is the net shortwave radiant flux, *L*_net_ is the net longwave radiant flux, *H*_S_ is the sensible heat flux, *H*_L_ is the latent heat flux, *H*_R_ is the heat flux associated with rainfall, *H*_G_ is the subsurface conductive heat flux, and *M* is the surface melt energy (0 W m^−2^ when the surface temperature is less than 273.15 K). These fluxes are defined as positive when they are directed into the snow/ice surface.

## Code Availability

The NHM-SMAP source code is available subject to a licence agreement with the Meteorological Research Institute, the Japan Meteorological Agency. Any researchers interested in the code are encouraged to contact the corresponding author (Masashi Niwano, mniwano@mri-jma.go.jp), who will assist them in obtaining a licence (by signing a contract) for the code.

## Supplementary information

Supplementary Tables and Figures

## Data Availability

All NHM–SMAP model output data presented in this study are available upon request by contacting the corresponding author (Masashi Niwano, mniwano@mri-jma.go.jp).
