# Supplementary material for: Cloud-driven modulations of Greenland ice sheet surface melt
Source: Sci Rep. 2019 Jul 17;9:10380. doi: 10.1038/s41598-019-46152-5 (PMC6637179; doi:10.1038/s41598-019-46152-5)
Supplement: Supplementary file 1 — Supplementary Tables and Figures [file 41598_2019_46152_MOESM1_ESM.pdf]

Supplementary information

Cloud-driven modulations of Greenland ice sheet surface melt

Masashi Niwano[1], Akihiro Hashimoto[1], and Teruo Aoki[2, 1]

[1] **Meteorological Research Institute, Japan Meteorological Agency, Tsukuba, 305-0052 Japan**

[2] **National Institute of Polar Research, Tachikawa, 190-8518 Japan**

Supplementary Table 1 | 2012–2013 area-averaged cloud fraction and cloud effects on the ice sheet SEB, SMB, and surface meteorological conditions simulated by NHM-SMAP. *P*, *T*, and *q* represent surface pressure, 2 m air temperature, and 2 m water vapour mixing ratio (w.e. means "water equivalents"). All values except for those for cloud fraction were obtained by subtracting clear-sky simulation results from all-sky simulation results. Each result is area-averaged over the entire ice sheet as well as the low-elevation (<1000 m a.s.l.) ablation areas of the southern and western regions of the ice sheet (see Fig. 1) during March–May 2013 (1213_MAM), June–August 2013 (1213_JJA), and September 2012 to August 2013 (1213).

	Entire ice sheet			Southern and western ablation areas		
	1213_MAM	1213_JJA	1213	1213_MAM	1213_JJA	1213
Cloud fraction	0.48	0.45	0.51	0.42	0.40	0.45
CRE (W m^{-2})	16.7	18.6	19.6	16.8	4.6	17.8
ΔS_{net} (W m^{-2})	–4.8	–10.3	–4.2	–6.7	–19.5	–7.4
ΔL_{net} (W m^{-2})	21.5	28.9	23.8	23.4	24.1	25.2
ΔH_{S} (W m^{-2})	–0.9	0.3	–1.6	3.9	1.1	2.4
ΔH_{L} (W m^{-2})	–3.9	–7.6	–5.2	–9.5	–17.3	–12.8
ΔM (W m^{-2})	0.0	–1.1	–0.3	–0.5	–13.7	–3.3
ΔRU (mm w.e. day^{-1})	0.00	–0.45	–0.09	–0.08	–4.44	–0.88
$\Delta \mathrm{SU}_{s}$ (mm w.e. day^{-1})	0.02	0.10	0.04	0.17	0.34	0.24
$\Delta \mathrm{SU}_{ds}$ (mm w.e. day^{-1})	0.04	0.02	0.03	0.15	0.01	0.05
ΔP (hPa)	–0.2	–0.2	–0.2	–0.5	–0.3	–0.6
ΔT (K)	2.2	1.5	2.3	0.5	–0.1	0.6
Δq (g kg^{-1})	0.0	–0.1	0.0	–0.2	–0.6	–0.3

Supplementary Table 2 | 2013–2014 area-averaged cloud fraction and cloud effects on the ice sheet SEB, and SMB, and surface meteorological conditions simulated by NHM-SMAP. Same as Supplementary Table 1 but for the 2013–2014 mass balance year.

	Entire ice sheet			Southern and western ablation areas		
	1314_MAM	1314_JJA	1314	1314_MAM	1314_JJA	1314
Cloud fraction	0.49	0.45	0.53	0.44	0.40	0.47
CRE (W m^{-2})	17.3	18.0	19.6	18.0	2.2	17.1
ΔS_{net} (W m^{-2})	–4.7	–10.6	–4.3	–7.3	–20.3	–7.7
ΔL_{net} (W m^{-2})	22.0	28.7	23.9	25.3	22.5	24.8
ΔH_S (W m^{-2})	–1.2	0.3	–1.7	2.5	0.5	1.7
ΔH_L (W m^{-2})	–3.6	–8.1	–5.1	–7.1	–19.6	–12.2
ΔM (W m^{-2})	0.0	–1.6	–0.4	–0.7	–17.5	–4.4
ΔRU (mm w.e. day^{-1})	–0.01	–0.57	–0.13	–0.24	–5.39	–1.25
ΔSU_s (mm w.e. day^{-1})	0.02	0.11	0.04	0.13	0.35	0.23
ΔSU_{ds} (mm w.e. day^{-1})	0.02	0.01	0.02	0.06	0.00	0.01
ΔP (hPa)	–0.2	–0.3	–0.2	–0.7	–0.3	–0.7
ΔT (K)	2.2	1.3	2.3	0.6	–0.1	0.6
Δq (g kg^{-1})	0.0	–0.1	0.0	–0.2	–0.7	–0.3

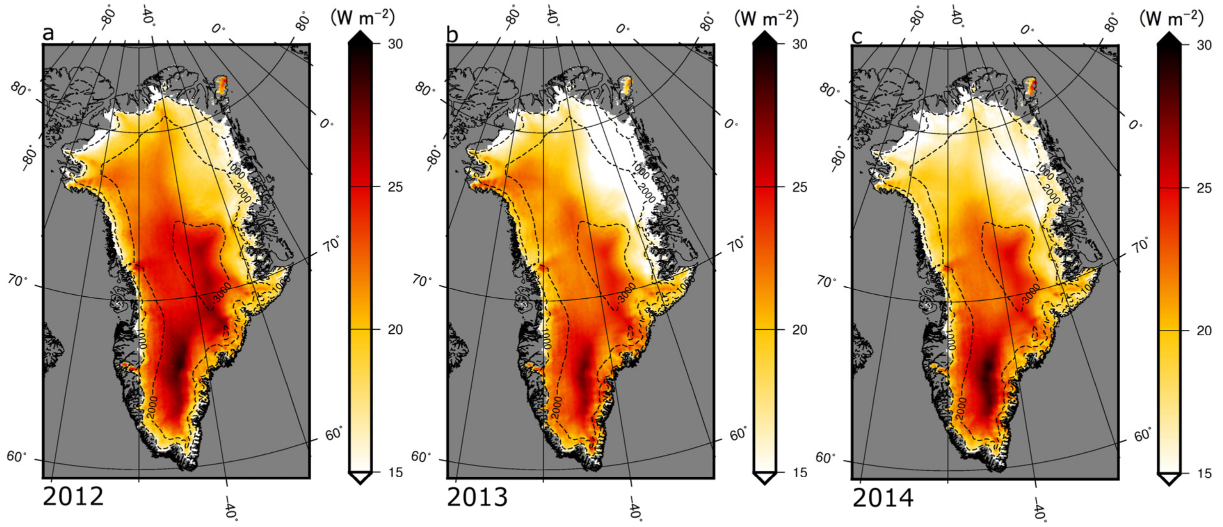

Supplementary Figure 1 | Annual average ice sheet cloud radiative effects simulated by NHM-SMAP. Annual average ice sheet cloud radiative effects for the **a** 2011–2012, **b** 2012–2013, and **c** 2013–2014 mass balance years. Contours on the ice sheet and peripheral ice caps indicate surface elevation (contour interval 1000 m).

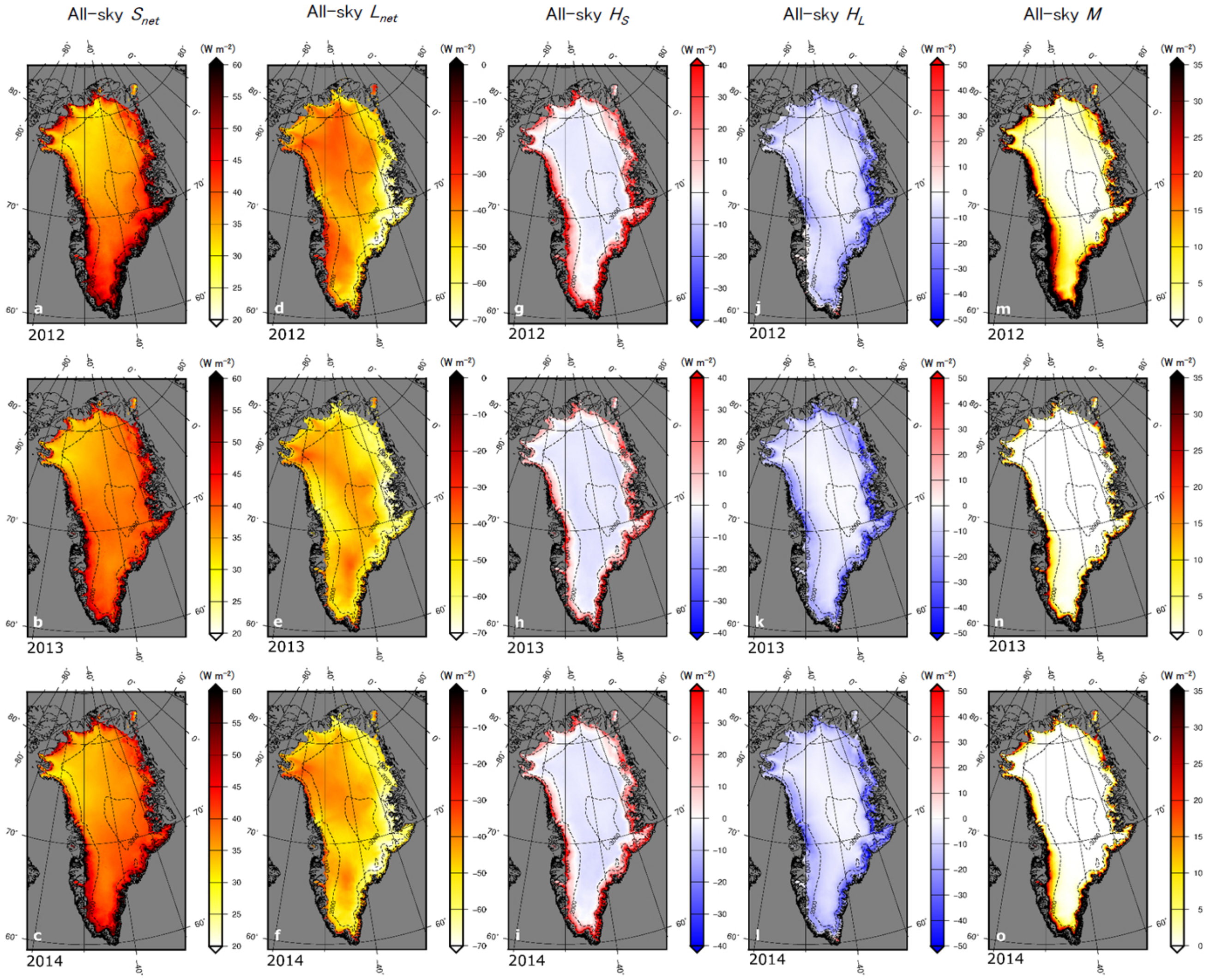

Supplementary Figure 2 | All-sky ice sheet SEB during JJA simulated by NHM-SMAP. a, **b**, **c** Net shortwave radiant flux; **d**, **e**, **f** net longwave radiant flux; **g**, **h**, **i** sensible heat flux; **j**, **k**, **l**, latent heat flux; and **m**, **n**, **o** surface melt energy in 2012, 2013, and 2014, respectively.

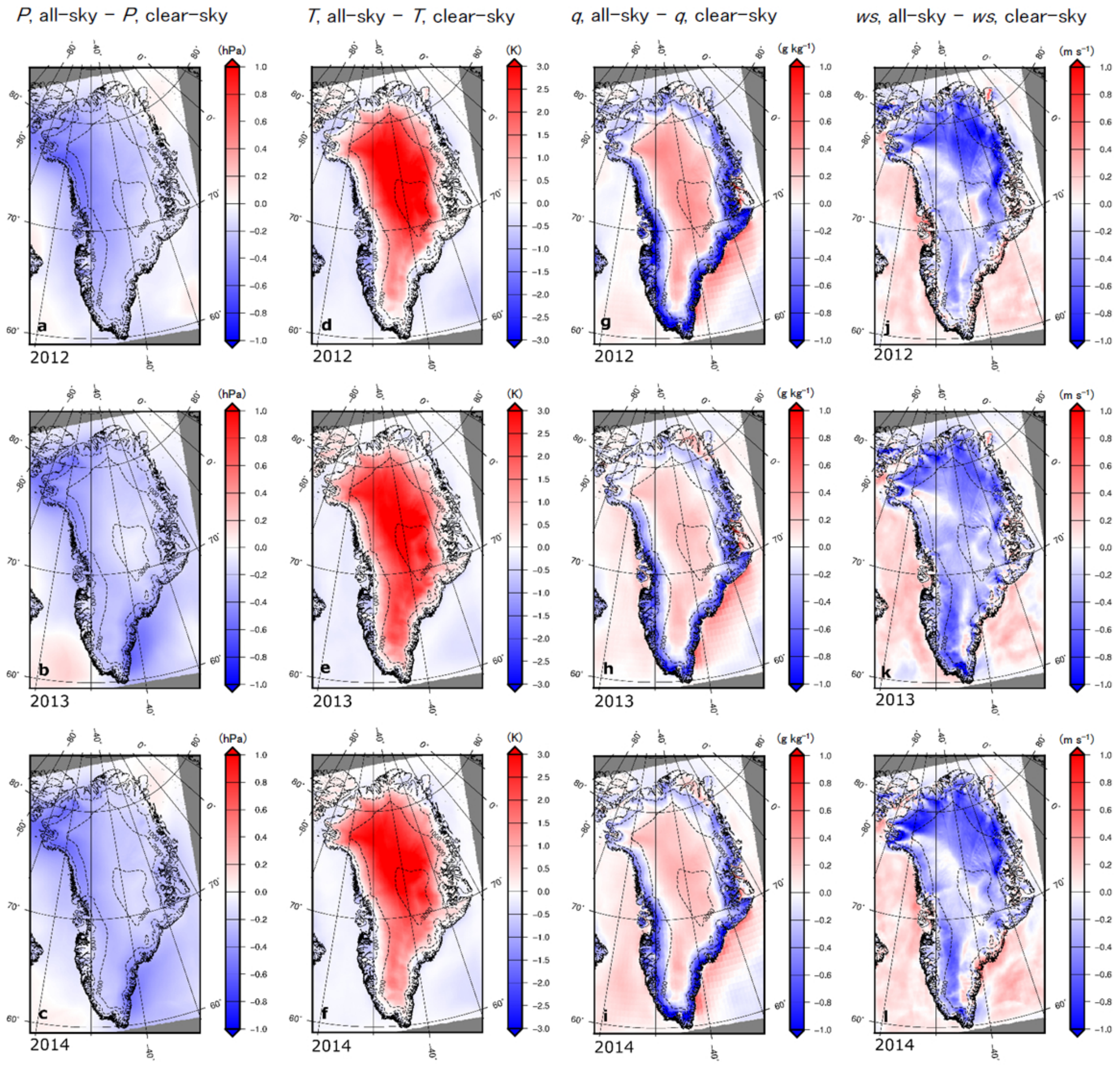

Supplementary Figure 3 | Impacts of clouds on surface meteorological conditions around the ice sheet during JJA simulated by NHM–SMAP. Changes in **a**, **b**, **c**, surface pressure (*P*); **d**, **e**, **f**, the 2 m air temperature (*T*); **g**, **h**, **i**, the 2 m water vapour mixing ratio (*q*); and **j**, **k**, **l**, the 2 m wind speed (*ws*) caused by the presence of clouds in 2012, 2013, and 2014, respectively. All values were obtained by subtracting clear-sky simulation results from all-sky simulation results.

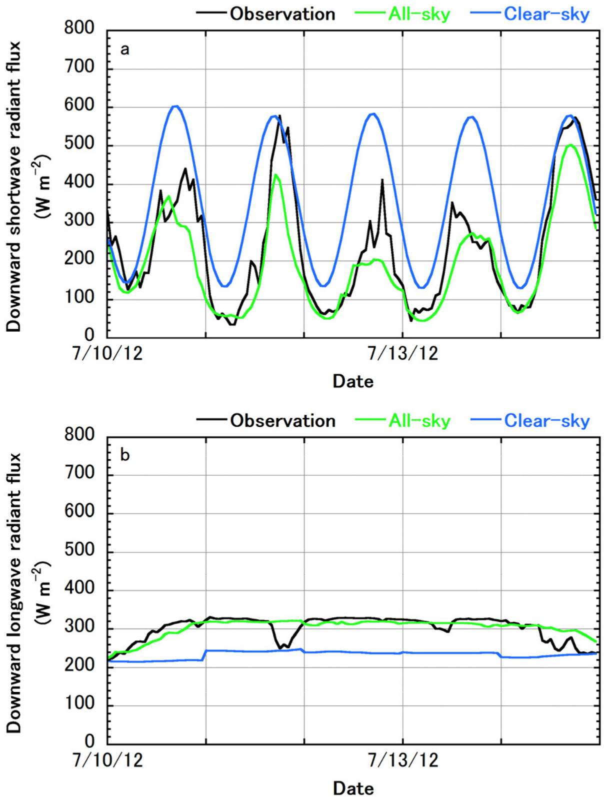

Supplementary Figure 4 | Observed and simulated downward shortwave and longwave radiant fluxes at the SIGMA-A site from 10 to 15 July 2012. Downward (**a**) shortwave and (**b**) longwave radiant fluxes. The position of the SIGMA-A site is indicated in Fig. 1d.
